# Correlation of sonographically-determined residual urine volume with lower urinary tract symptoms in adult males at a tertiary hospital

**DOI:** 10.4314/gmj.v58i2.4

**Published:** 2024-06

**Authors:** Linda Nketiah, Klenam Dzefi-Tettey, Raphael N Mayeden, Ambrose Agborli, Benard Ohene-Botwe, Yaw B Mensah

**Affiliations:** 1 Department of Diagnostics and Imaging, University of Ghana Medical Centre, Ghana; 2 Department of Radiology, Korle Bu Teaching Hospital, Accra, Ghana; 3 Department of Radiology, School of Medicine, University of Health and Allied Sciences, Ho, Ghana; 4 Department of Statistics and Actuarial Sciences, University of Ghana; 5 Department of Midwifery & Radiotherapy, School of Health and Psychological Sciences, City, University of London, London, UK; 6 Department of Radiology, University of Ghana Medical School, Legon, Ghana

**Keywords:** post-void residual urine, lower urinary tract symptoms, feeling of incomplete emptying, international prostate symptom score, Quality of life

## Abstract

**Objective:**

To determine the correlation between the severity of LUTS as measured by the International Prostate Symptom Score (IPSS) and PVR urine volume measured by transabdominal ultrasound in patients with LUTS, and to determine the correlation between ‘feeling of incomplete bladder emptying and sonographically measured PVR urine volume.

**Design:**

Correlational cross-sectional study

**Setting:**

Ultrasound Unit of the Radiology Department and Urology Clinic of Korle Bu Teaching Hospital

**Participants:**

Male patients (n=256) aged 40 years or older who presented to the urology department of Korle Bu Teaching Hospital with LUTS and gave their written consent were enrolled. The presence and severity of LUTS were evaluated using the IPSS. The PVR urine was measured using a real-time transabdominal ultrasound scan.

**Main outcome measure:**

Severity of LUTS and Residual urine volume

**Results:**

The mean PVR urine volume was 84.5ml. Most respondents (57.3%, n=146) had PVR urine volume below 50ml, with 27% (70 patients) having PVR urine volume above 100ml. PVR urine volume and total IPSS showed no statistically significant correlation. All age groups of respondents scored above 4 for Quality of life (QoL). ‘Intermittency’ is the IPSS symptom, which showed a statistically significant correlation with PVR urine volume. PVR urine volume did not correlate statistically with the ‘feeling of incomplete emptying’.

**Conclusions:**

There was no statistically significant correlation between the total IPSS and PVR urine volume. Thus, residual urine volume does not correlate with the severity of LUTS. The ‘feeling of incomplete emptying’ does not correlate with PVR urine volume.

**Funding:**

None declared

## Introduction

The International Continence Society (2015) defines LUTS as symptoms that result from conditions affecting the bladder and the urethra. LUTS have multifactorial causes, thus presenting a challenge to managing this problem. Despite various options for medical treatments, lower urinary tract obstruction with its associated symptoms remains a significant economic burden and public health problem.^1^

A review by Zhang et al. 2 found that LUTS were highly prevalent globally and estimated to affect 2.3 billion people in 2018, with 44.7% being men. In Ghana, Chokkalingam et al. in 2011^3^ reported the prevalence of moderate-to-severe LUTS (IPSS⩾8) to be 19.9%.

These symptoms negatively affect affected individuals' Quality of life (QoL).^4^ The prevalence of LUTS generally increases with increasing age in men. Compared to younger men, men over 80 are more likely to have LUTS and more likely to complain of incomplete emptying, frequency, urgency, and weak stream.^5^ LUTS can be distressing for affected patients. Some individuals with LUTS organise their daily activities based on the availability of a toilet facility/restroom.

LUTS can be evaluated using a questionnaire such as the International Prostate Symptom Score (IPSS) to establish the impact of these symptoms on a patient's routine. The IPSS is recognised globally as a tool for assessing LUTS severity.^6^ Although initially adopted for the clinical diagnosis of benign prostatic hyperplasia (BPH), the IPSS is now commonly used as a more specific measure of LUTS irrespective of the pathology in men and women^7^. It is intrinsically consistent, sensitive to changes in symptomatology, and has good test-retest reliability.^8^ It is designed to be self-administered by patients; however, the inability to read and write can be a major drawback in administering the questionnaire.^9^,^10^

The IPSS consists of seven questions encompassing storage, voiding, and post-micturition symptoms and an eighth question on QoL.^8^ The feeling of incomplete emptying assessed by the first question of the IPSS can be defined as “complaint that the bladder does not feel empty after micturition”.^11^ This symptom is associated with lower QoL^12^ and worsening of voiding and storage symptoms.^13^

A feeling of incomplete emptying does not always correlate strongly with the measured PVR urine volume.^13^ A study by Sountoulides et al. 14 reported that incomplete bladder emptying does not always correlate with the finding of significant residual urine volume and can even be felt with an empty bladder. Cayetano-Alcaraz et al in 2016^15^ documented similar findings.

Measurement of PVR urine, the amount of residual urine in the bladder after a voluntary void^16^, is a screening test for evaluating voiding dysfunction. Like uroflowmetry, the measurement of PVR is an effective tool that helps identify patients needing further evaluation and treatment follow-up. Threshold values for an abnormal PVR are poorly defined, but most urologists agree that volumes of 50 mL to 100 mL constitute the lower threshold, which defines abnormal residual urine volume.^17^

There are two methods of measuring PVR urine: sterile catheterisation and bladder ultrasound. Urethral catheterisation has been accepted as the gold standard for PVR urine measurements, but this is a source of discomfort to the patient and carries a risk of urinary tract infection and urethral trauma.^18^ The bladder ultrasound, on the other hand, is a non-invasive, timesaving, widely available, and well-tolerated method for measuring PVR urine.^17^ Due to its non-invasiveness, there is no risk of urethral injury, and it can be repeated as often as needed to follow up on the patient's response to management, thus the preferred method.

Traditionally, ultrasound estimation of bladder volume can be performed in two ways: real-time ultrasound to visualise the bladder directly 8, 19, 20 or a portable bladder scanner to calculate the volume automatically without directly visualising the bladder.^21^ Most ultrasound machines have a function to automatically calculate volumes from the direct measurements of the anteroposterior, transverse, and craniocaudal dimensions of the bladder. Referring urologists and general practitioners in Ghana often request PVR urine volume, but there has not been documented data on how this volume correlates with the patient's LUTS severity.

Significant chronic residual urine has been shown to lead to elevated intravesical pressures, which may lead to hydroureteronephrosis and, eventually, renal failure.^22^ Patients with significant PVR urine will, therefore, require close follow-up to know when to initiate medical or surgical therapy. Very importantly, bothersome LUTS, despite insignificant PVR urine, also need attention due to the great impact on QoL. For these reasons, urologists often want to know the PVR urine volume in patients with LUTS. Unfortunately, there is limited data in Ghana regarding the relationship between PVR urine and the severity of LUTS and the connection between the ‘feeling of incomplete bladder emptying’ and the measured PVR urine. This study aims to determine the correlation between the severity of LUTS, as assessed by the IPSS, and PVR urine volume, measured by transabdominal ultrasound in patients with LUTS. Additionally, it aims to establish the correlation between the feeling of incomplete bladder emptying and sonographically measured PVR urine volume.

## Methods

### Study Design and Setting

The study was a cross-sectional study conducted in the ultrasound unit of the Radiology Department with subjects from the Urology Clinic of the Korle Bu Teaching Hospital from August 2020 to June 2021. The Korle Bu Teaching Hospital is the largest tertiary hospital in Ghana, having the highest concentration of urologists and, thus, receiving referrals from most parts of the country. With an annual Urology outpatient average attendance of about 12500 patients, the urology clinic gets a fair number of patients with LUTS (average of 7180 cases of LUTS per annum). The Radiology Department of the Korle Bu Teaching Hospital has a busy ultrasound unit with individual scanning suites and a built-in toilet facility.

### Sample size

Using a sample size formula proposed by Charan and Biswas for cross-sectional studies,^23^


$$N=\frac{{Z_{\left(1-a/2\right)}}^{2}\;p\;(1-p)}{d^{2}}$$

Where N= sample size estimate of patients with lower urinary tract symptoms

Z_(1-a/2)_ = Standard normal variate (at 5% type 1 error (p<0.05), it is 1.96.

Using a prevalence of 19.9 % per the study by Chokkalingam et al. in 2011, the calculated minimum sample size was 245.

Assuming a 10% participant attrition, the estimated sample size comprised 270 respondents.

### Study participants

Male patients aged 40 years or more who presented to the urology outpatient department of the Korle Bu Teaching Hospital with lower urinary tract symptoms and gave their written consent were enrolled in the study. Patients with LUTS who had a history of previous urological (bladder/prostate) surgery or urethral/suprapubic catheter in-situ at the time of study, those with diabetes mellitus, history of urinary tract infection three months before presentation, known neurological disease which can affect sensation, or on diuretic medications were excluded.

### Data collection

Using convenience sampling, patients who met the eligibility criteria and consented to participate in the study were consecutively recruited according to their availability until the required sample size was obtained. The investigator obtained written informed consent, after which a pre-tested questionnaire, which consisted of demographic characteristics and the IPPS survey questions, was used to collect the data. The principal investigator performed All PVR urine scans using a curvilinear transducer frequency of 3.5 MHz and a volume estimator of an Edan U60 ultrasound machine.

### Procedure for sonographic determination of postvoid volume

Just before the ultrasound scan was performed, subjects were instructed to empty their bladder as completely as possible in a private room. The participant was positioned supine, and the area between each participant's umbilicus and pubic hairline was uncovered. It was prepped with acoustic/ultrasound gel, and the transducer was placed at approximately 3 cm superior to the symphysis pubis so that the beam would point toward the expected bladder location. The bladder was scanned in axial (transverse) and sagittal planes, with sweeps through the bladder in an arc. The transducer was swept from the cephalad to the caudad in the axial plane, angling to include the whole bladder. In the sagittal plane, the transducer was initially placed in the midline and then swept in an arc to both sides to ensure that the entire bladder was visualised. The greatest transverse dimension on the transverse view, as well as the anteroposterior and superior-inferior dimensions on the sagittal view, were determined with the ultrasound calliper. The ultrasound calliper was placed in the inner wall of the bladder during the measurement of the urine volume, thus excluding the bladder wall.

The residual urine volume in this study was determined using the ultrasound machine's internal volume calculations. Two separate bladder ultrasound volume measurements were obtained. The average of the two measurements obtained was then recorded as the final PVR urine volume for that individual (this was done to minimize intra-observer variability).

### International Prostate Symptom Score (IPSS)

The IPSS questionnaire consists of eight items, which include seven 6-point scale questions on the LUTS (feeling of incomplete emptying, urinary frequency, interrupted stream, urinary urgency, weak urinary stream, urinary hesitancy/straining, and nocturia) and one 7-point scale question on patients' satisfaction with their urinary condition. Based on the criteria, symptom severity was divided into three groups: mild (a symptom score of 0–7), moderate (8–19), and severe (20–35).^8^

Seven grades represent the QoL or level of satisfaction of LUTS patients: “No problem” (0 point = Very satisfied/Delighted), “I'm all right” (1 point= pleased), “Somewhat satisfied” (2 points= mostly satisfied), “Half-satisfied, half-dissatisfied” (3 points= mixed), “Somewhat dissatisfied” (4 points=mostly dissatisfied), “Distressed” (5 points= unhappy), and “I can't stand it” (6 points =Terrible/Very dissatisfied).

### Statistical analysis

Data was analysed using Microsoft Excel 2016 and IBM Statistical Package for the Social Sciences (SPSS) software version 20. The statistical significance level was set at p < 0.05. A non-parametric Spearman's Correlation test was used to assess the level of association between the data variables since the data was found not to follow a normal distribution. As a result, median values were reported alongside the mean values.

Also, one-way non-parametric ANOVA (Kruskal-Wallis test) with post hoc analysis in SPSS was used to compare variables.

### Ethical Considerations

Ethical approval was obtained from the Scientific and Technical Committee and Institutional Review Board of the Korle Bu Teaching Hospital (Ref: KBTH-STC 000022/2020 and KBTH-IRB/00022/2020 respectively). Informed consent was obtained from each participant.

## Results

### Demographic information

After discarding incompletely filled questionnaires, 256 males aged 40 and above participated in this study. The ages of the subjects enrolled in this study ranged from 40-83 years, with a mean of 65.1 ± 8.4 years old. The median age range was 60-69 years (with a median age of 65.0 years). The majority of the subjects were in the 60-69 years range. Table 1 shows the demographic data of respondents.

**Table 1:** Demographic characteristics of subjects

Variable	Values
Age (Years)	
Minimum	40
Maximum	83
Mean ± SD	65.1 ± 8.4
**Occupational status (Count (%))**	
Retired	161 (62.9)
Active Service	95 (37.1)
**Educational level (Count (%))**	
None	7 (2.7)
Primary	76 (29.7)
Secondary	111 (43.4)
Tertiary	39 (15.2)
Postgraduate	23 (9.0)

As shown in Table 1, the majority (n=161, 62.9%) of respondents recruited for this study had retired from active service, and 37.1% (n=95) were in active service. Secondary/middle school leavers (n=111, 43.4%) formed the majority of the respondents, with those having no formal education (n=7, 2.7%) being the least.

The mean duration of symptoms was 3.8±4.6 years (median -2 years), with the shortest duration being 0.1 year (1 month 2 weeks) and the longest duration being 20 years. Younger respondents had shorter symptom durations.

### Residual urine volume

The data for the post-void residual urine volume was skewed. The mean, standard deviation and median values were 84.5 ml, 112.6 and 49 ml, respectively with the least recorded residual urine being 0 (complete bladder emptying) and the largest volume being 669 ml. Most respondents (57.3%, n=146) had PVR urine volume below 50 ml, 15.7% (n=40) between 51 and 100 ml and 27.0 % (n=70) had volumes above 100 ml

### IPSS and QoL

The observed statistics (mean, standard deviation, median and range values) across all the patients were: Total IPSS score (Mean ± SD = 16.3 ± 6.4; median = 17; range = 4-31); QoL on a scale of 1-6 (Mean ± SD = 4.4 ± 1.2; median = 5; range = 1-6), shown in Table 2. Of the 7 LUTS assessed by the IPSS, the symptom with the highest mean score was nocturia, with a mean score of 3.2 ± 1.4 and a Median of 3.

**Table 2:** IPSS and the observed characteristics

Parameters	Mean± SD	Range		Median
		min	max	
Incomplete emptying	2.2±1.7	0	5	2.0
Frequency	2.6±1.5	0	5	2.0
Intermittency	2.2±1.7	0	5	2.0
Urgency	1.9±1.7	0	5	2.0
Weak stream	2.4±1.7	0	5	2.5
Straining	1.8±1.8	0	5	1.0
Nocturia	3.2±1.4	0	5	3.0
Total IPSS score	16.4±6.5	4	31	17.0
QoL	4.4±1.3	1	6	5.0
PVR	84.5±112.6	0	669	49.0

Min=Minimum; Max = Maximum; SD = Standard deviation

The mean QoL score across all age groups was above 4, irrespective of their total IPSS score or residual urine volume; the age group with the highest mean QoL score of 5 was the 40-49 age range. The age group with the highest PVR was the 70-79 years group, with a mean PVR urine of 96.5ml (Median = 45.5 ml). The 70-79 years group also recorded the highest mean duration of symptoms of 4.9 ± 4.8 years (Table 3).

**Table 3:** Age groupings and Duration of symptoms, IPSS and PVR urine volume

Age range (years)	Mean duration of symptoms score ± SD (range) in years/Median	Mean Total IPSS Score ± SD (range)/Median	Mean QoL Score ± SD (range)/Median	Mean PVR urine volume Score ± SD (range)/Median
40-49	0.1± 0.0 (0.1-0.1)/ 0.1	7.25 ± 0.5 (7-8)/ 7.0	5 ± 0.0 (5-5)/ 5.0	54 ± 2.7 (50-56)/ 55.0
50-59	1.8 ± 1.7(0.1-5)/ 0.9	17.7 ± 7.5 (4-29)/ 20.0	4.2 ± 1.4 (1-6)/ 5.0	74.0 ± 125.0 (0-669)/ 45.0
60-69	4.1 ± 4.7 (0.1-20.0)/ 2.0	16.0 ± 5.9 (5-30)/ 16.0	4.4 ± 1.4 (1-6)/ 5.0	84.1 ± 98.1 (0-526)/ 51.0
70-79	4.9 ± 4.8 (0.1-20)/ 5.0	17.0 ± 6.3 (5-31)/ 16	4.5 ± 1.1 (2-6)/ 5.0	96.5 ± 133.7 (7-560)/ 45.5
80-83	4.8 ± 7.9 (0.2-20)/ 0.5	13.6 ± 6.4 (8-24)/ 9.0	4.7 ± 0.4 (4-5)/ 5.0	78.8 ± 65.3 (22-200)/ 50.0

Key: SD = standard deviation; QoL = Quality of life on a scale of 1-6 (1-Delighted, 6-Terrible); IPSS=International Prostate Symptom Score; PVR = postvoid residual.

The majority of respondents (57 %, n=146) had severe LUTS. This category also scored high for QoL (poor Quality of life). Those with severe symptoms recorded higher PVR urine than those with mild and moderate symptoms. Mild IPSS showed a statistically significant correlation with PVR urine volume [r(254)=.90,p=.029], and the QoL r(254)=.64,p=.002]. However, moderate r(254)=.37,p=.001], and severe r(254)=.50,p=.001] IPSS showed a statistically significant correlation with QoL but not with PVR urine { moderate r(254)=.15, p=.070]; severe r(254)=.05, p=.652]}

There was a statistically significant positive correlation between PVR urine volume and duration of symptoms (rs= 0.130, p = 0.038) and intermittency (rs= 0.212, p = 0.001). Also, the QoL score and total IPSS demonstrated a statistically significant positive correlation (rs= 0.541, p = 0.001). However, PVR urine volume did not show a statistically significant correlation with frequency of micturition, incomplete emptying, voiding urgency, straining, weak stream, nocturia, age, QoL and total IPSS (Table 5).

**Table 5:** Correlation of PVR urine volume with individual IPSS, total IPSS, QoL and other variables

Test components	Correlation (*r*_*s*_-value)	*p-value*
Frequency of micturition vs PVR urine volume	0.015	0.805
Incomplete emptying vs PVR urine volume	0.079	0.209
Urgency vs PVR urine volume	0.057	0.361
Straining Vs PVR urine volume	-0.098	0.118
Weak stream vs PVR urine volume	0.006	0.928
Intermittency vs PVR urine volume	0.212	0.001*
Nocturia vs PVR urine volume	-0.111	0.076
Duration of symptoms	0.130	0.038*
Age vs PVR urine volume	0.085	0.177
QoL score vs Total IPSS	0.541	0.001*
QoL vs PVR urine volume	0.038	0.549
Total IPSS vs PVR urine volume	0.085	0.176

* statistically significant

## Discussion

This study evaluated 256 respondents aged 40 years and above. The mean age of respondents was 65.1±8.4 years, and the 60-69 age range was the most represented (45.7%, n=117). These findings support the fact that LUTS is common among older adults.^5^

The mean duration of symptoms was 3.8 (Median = 2 years). The reported duration of symptoms was wide, between a few months and 20 years. The disparity could be attributed to many factors, such as the insidious way LUTS present and the general perception that voiding symptoms are part of the ageing process. Studies elsewhere have shown that many elderly males seek professional help only when their inconvenience exceeds tolerance level.^25^,^26^

Younger respondents had shorter symptom durations than older respondents, with the shortest mean symptom duration (6 weeks) noted in the 40-49 years range, followed by the 50-59 years range. These age ranges comprised individuals who were in active service; thus, the impact of LUTS on their daily activities/work schedule was deemed significant interference (impaired Quality of life), resulting in their relatively earlier presentation to the hospital. Ojewola et al. 27 reported that one of the most common reasons for men with LUTS seeking help was impaired Quality of life, supporting the fact that impaired Quality of life is a push factor for early presentation to the hospital.

Most respondents (57.3%, n=146) had PVR urine below 50ml, with 27% having PVR more than 100ml. Lammers et al. 28, in a similar study, also reported that 27% of respondents recorded PVR urine greater than 100ml. Although no numerical value of PVR has been universally accepted into current clinical practice^16^, a study by Kelly^17^ considered PVR volume of less than 50 ml adequate bladder emptying in young adults and between 50 and 100 ml in older people. In the study by Lammers et al. 28, residual urine volume of more than 100 mL was considered abnormal. Thus, most of the respondents in our research can be deemed to have adequate bladder emptying.

A statistically significant positive correlation was seen between PVR urine and duration of symptoms. Thus, patients who have had LUTS for longer periods recorded higher PVR urine, supporting the assertion that LUTS, if left untreated over a period, can result in urinary retention^29^ with its attendant increased residual urine volume. In managing patients with LUTS, careful attention to the severity of symptoms and their impact on the Quality of life of the affected individual is of the utmost importance. The mean IPSS in the index study was 16.3, with nocturia being the symptom with the highest score. The results compared favourably to what was documented by Lammers et al.^28^ The mean IPSS was similar (16.5), but the weak stream was the symptom most participants of the Lammers et al. study mentioned: “almost always occurs.”

Significant LUTS is usually defined as a total IPSS of 8 and above.^30^ This comprises the group with moderate and severe IPSS scores. In this study, most respondents (57%, n=146) had severe LUTS, and a good proportion (34.8%, n=89) had moderate LUTS. A smaller percentage of respondents (8%, n= 21) had only mild LUTS. In a study by Hamid et al. 31 in men with LUTS, most respondents (50%) had severe symptom scores, similar to the findings in the index study. Awaisu et al. 32 also reported that most respondents (55%) had moderate symptom scores. The low numbers for those with mild symptoms may be because most people do not seek medical attention when they have mild disease. Therefore, it can be inferred that most patients who present to the hospital for evaluation of LUTS have clinically significant symptoms and must be given prompt attention. Ojewola et al. 27 corroborated this finding in their study, which reported moderate-to-severe symptoms as one of the most common reasons why men with LUTS seek help.

In this study, the mean PVR urine for those with mild, moderate and severe IPSS was 52ml, 75.3ml and 107ml, respectively. Among these three categories of severity grading, only mild IPSS showed a statistically significant correlation with the measured PVR urine volume. This was slightly different from the findings by Aldamanhori^33^ in Saudi Arabia. In that study, the PVR urine measurement showed no statistical difference in all three categories of symptom severity. The conclusions of both studies support the point that factors other than symptom severity, such as urinary effort and total score for voiding symptoms, influence the residual urine volume.^15^

Although respondents with moderate and severe IPSS recorded relatively higher mean PVR urine, this was not found to be statistically significant. There was no statistically significant correlation between the total IPSS and PVR urine volume. Previous studies have shown inconsistent results on the correlation of IPSS with residual urine volume. Although a few studies^34^,^35^ have reported a correlation between IPSS and post-void residual urine volume, others like that by Lammers et al. 28 did not find a relation between the IPSS score and an abnormal residual urine volume in men aged over 50 with LUTS consulting primary care. A possible reason for the differing findings between various studies could be the varying study settings, with some studies being community-based and others being hospital-based or primary care settings.

Out of the seven symptoms assessed by the IPSS questionnaire in this study, ‘intermittency’ showed a statistically significant correlation with PVR. The other 6 LUTS (including ‘feeling of incomplete emptying’) assessed by the IPSS questionnaire in this study showed no statistically significant correlation with the measured PVR urine. However, this study found that the feeling of incomplete emptying also impaired QoL.

Cayetano-Alcaraz et al. 15 and Aldamanhori^33^ reported similarly that the sense of incomplete emptying showed no statistically significant correlation with the measured PVR urine.

Ozlulerden et al. 36 investigated the association between PVR and the feeling of incomplete emptying in both men and women. Interestingly, they found a positive correlation between the feeling of incomplete bladder emptying and PVR volume in all age groups of women. Still, in men, such a relationship was found only in those above 60 years. The findings in the Ozlulerden et al. study could be attributed to the fact that the mean post-void residual urine in those with and without the complaint of incomplete emptying did not differ much in the age group below 60 years in men.

Regarding Quality of life (QoL)/border score and IPSS, there was a statistically significant positive correlation between total IPSS and QoL. In a study on LUTS among middle-aged and elderly Nigerian men, Adegun et al. 37 concluded that there is no severity of LUTS in which patients' QoL is not impaired. In our study, those with moderate and severe IPSS recorded high bother scores; thus, it can be said that most patients with LUTS had their Quality of life affected to some degree.

### Limitations

The IPSS questionnaire was designed in English; thus, patients who could not read English had to be assisted by translation into a comprehensible language. This creates room for interpreter bias, influencing the LUTS severity assessment.

## Conclusion

The severity of LUTS, as evaluated by the IPSS, did not correlate with the measured PVR urine volume. Instead, the severity of the symptoms correlated well with the degree of QoL impairment. This study also showed that the feeling of incomplete emptying does not show a statistically significant correlation with the measured post-void residual urine volume. In this study, the IPSS symptom that correlated best with PVR urine volume was ‘intermittency’.

Although the severity of LUTS and PVR urine does not show a statistically significant correlation, it independently influences the choice of management, underscoring the importance of measuring and documenting the PVR urine in the abdominopelvic ultrasound report of all patients who present with LUTS. Those found to have significant PVR urine can be treated irrespective of the severity of the symptoms. Patients with bothersome symptoms despite insignificant residual urine will also benefit from medical therapy to improve their Quality of life.

## Figures and Tables

**Table 4:** Groupings of IPSS severity with QoL and PVR

Total IPSS score category	n (%)	Mean QoL ± SD/(median)	Mean PVR± SD/(Median)
Mild IPSS score (0-7)	21(8.2)	2.52±1.3/ (2)	52.38±24.9/ (50)
Moderate IPSS score (8-19)	89(34.8)	4.30±1.2/ (5)	75.27±91.7/ (48.5)
Severe IPSS score (20-35)	146(57.0)	4.96±0.9/ (5)	107.06 ±147.8/ (49)

n =number of respondents, % =percentage
